# Winter warming post floral initiation delays flowering via bud dormancy activation and affects yield in a winter annual crop

**DOI:** 10.1073/pnas.2204355119

**Published:** 2022-09-19

**Authors:** Xiang Lu, Carmel M. O’Neill, Samuel Warner, Qing Xiong, Xiaochao Chen, Rachel Wells, Steven Penfield

**Affiliations:** ^a^Department of Crop Genetics, John Innes Centre, Norwich NR4 7UH, United Kingdom

## Abstract

Winter annual life history is conferred by the requirement for vernalization to promote the floral transition and control the timing of flowering. Here we show using winter oilseed rape that flowering time is controlled by inflorescence bud dormancy in addition to vernalization. Winter warming treatments given to plants in the laboratory and field increase flower bud abscisic acid levels and delay flowering in spring. We show that the promotive effect of chilling reproductive tissues on flowering time is associated with the activity of two FLC genes specifically silenced in response to winter temperatures in developing inflorescences, coupled with activation of a BRANCHED1-dependent bud dormancy transcriptional module. We show that adequate winter chilling is required for normal inflorescence development and high yields in addition to the control of flowering time. Because warming during winter flower development is associated with yield losses at the landscape scale, our work suggests that bud dormancy activation may be important for effects of climate change on winter arable crop yields.

## Significance

In temperate climates many plant species use long-term detection of winter chilling as a seasonal cue. Previously the timing of flowering in winter annual plants has been shown to be controlled by the promotion of the floral transition by chilling, known as vernalization. In contrast, many temperate perennial species produce flower buds prior to winter and require winter chilling to break bud dormancy to enable bud break and flowering in the following spring. Here we show that flowering time in winter annuals can be controlled by bud dormancy and that in winter oilseed rape–reduced chilling during flower bud dormancy is associated with yield declines.

Winter annual phenology is common in temperate environments and requires repression of flowering until release by chilling, known as vernalization. In Brassicas and many angiosperms the vernalization requirement is determined by *FLOWERING LOCUS C* (*FLC*) expression (1). In the United Kingdom winter oilseed rape (WOSR) is sown in late summer. It undergoes vernalization in midautumn and floral development proceeds during winter (2). The closely related model species *Arabis alpina* will also undergo the floral transition under chilling conditions rather than in spring (3), while in *Arabidopsis thaliana* and *Arabidopsis halleri* vernalization is also completed in autumn or early winter (4–6), with *A. thaliana* beginning floral development in late autumn (*SI Appendix*, Fig. S1). Therefore, the phenology exhibited by *Brassica napus* appears very general in the family. *FLC* continues to play a role in flower and seed development (7–9), but the importance of *FLC* expression in reproductive tissues is unclear. Furthermore, in winter arable crops correlative studies have suggested a link between declining winter chilling and low yields (10, 11) through as yet unclear mechanisms. In WOSR a reduction in autumn chilling in the vegetative phase can delay the floral transition by up to 1 mo (2). However, flowering itself was only delayed by 1 wk, showing that processes after the floral transition are important for the timing of flowering. During early floral development increased chilling is associated with higher yields (11), suggesting a further, as yet unclear role for chilling during reproductive development.

## Results

**Fig. 1.**
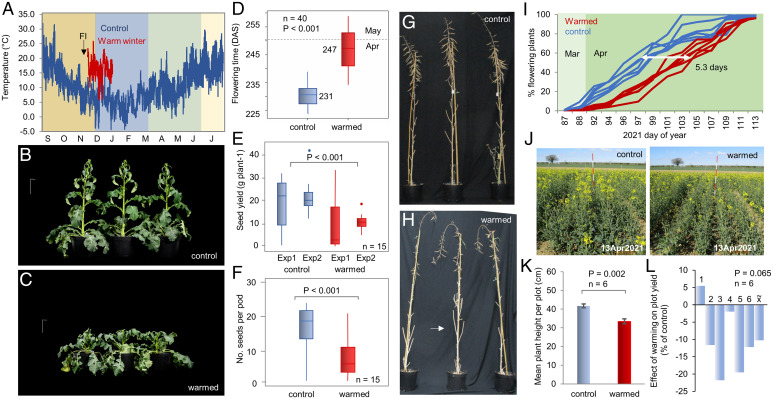
Winter warming during flower bud development delays flowering and is associated with yield reductions. (*A*) Temperature history for simulated 2016/2017 growing season in Norwich, UK (control) and the early winter warming treatment, relative to floral initiation (FI). (*B* and *C*) Representative control and warmed plants on simulated March 25, 2017. (*D*) Timing of first flowering in control and warm-treated WOSR, expressed in days after sowing (DAS). (*E* and *F*) Pod and seed set on warmed and control plants in two independent 11-mo simulation experiments. *P* value is derived from a one-way (*F*) or gwo-way (*E*) ANOVA. (*G* and *H*). Representative images of flowering plants showing reduced pod set in warmed plants. (*I*–*K*) Field warming delays bolting and flowering in WOSR. (*I*) First flowering time of individual warmed and control plots in March to April 2021. (*J* and *K*) Field warming delays bolting. (*L*) Effect of warming on yield in six plot pairs. *P* value was calculated by paired *t* test.

To investigate the effect of early winter warming on WOSR reproductive development, we used controlled environment rooms (CERs) programmed to reproduce the temperature and photoperiod of the 2016 to 2017 growing season in Norwich, UK, using data collected alongside a previous WOSR field trial (2) for which we had detailed morphological and molecular data (see *Materials and Methods*). Plant development in the seasonal simulation closely tracked previous field observations, including the timing of floral transition at the apex in the first week of simulated November (*SI Appendix*, Fig. S2*A*). Thus field phenology can be reproduced under controlled conditions. We then used a second CER to give a mean 10 °C warming treatment starting 1 wk after the floral transition, bringing plants to seed set together in simulated 2017 winter, spring, and summer (Fig. 1*A*). Previous work suggests that after vernalization warm temperatures should promote flowering through the ambient temperature pathway (12–14). However, WOSR plants warmed in winter did not resume growth (*SI Appendix*, Fig. S2*B*); instead, we found that bolting and flowering were delayed by 14 d on average by winter warming of floral buds (Fig. 1 *B*–*D*). Furthermore, in agreement with a previous correlative study of UK farm yields (11), seed yield per plant was significantly reduced in two independent experiments (Fig. 1 *E* and *F*), caused by fewer set pods and fewer seeds per pod in warmed plants (Fig. 1 *G* and *H*). This was accompanied by an increase in the frequency of abnormal flower buds on warmed plants, including bud abscission and asynchronous development of floral organs (*SI Appendix*, Fig. S3).

We also used a field plot warming system (2) to warm individual field trial plots in early winter in Norwich, UK, which on average delivered a mean 5.7 °C temperature gain for 4 wk (*SI Appendix*, Fig. S4). Field warming during early reproductive development also delayed bolting and flowering in WOSR confirming the existence of a previously unknown mechanism during reproductive development during which warm temperatures delay development (Fig. 1 *I*–*L*). In a paired plot design, we found an overall yield reduction in five of six pairs warmed versus control plots, which was weakly significant (*P* = 0.065; Fig. 1*L*). Taken together, our data show that winter chilling after the floral transition accelerates the reproductive development of WOSR and is associated with yield gains.

**Fig. 2.**
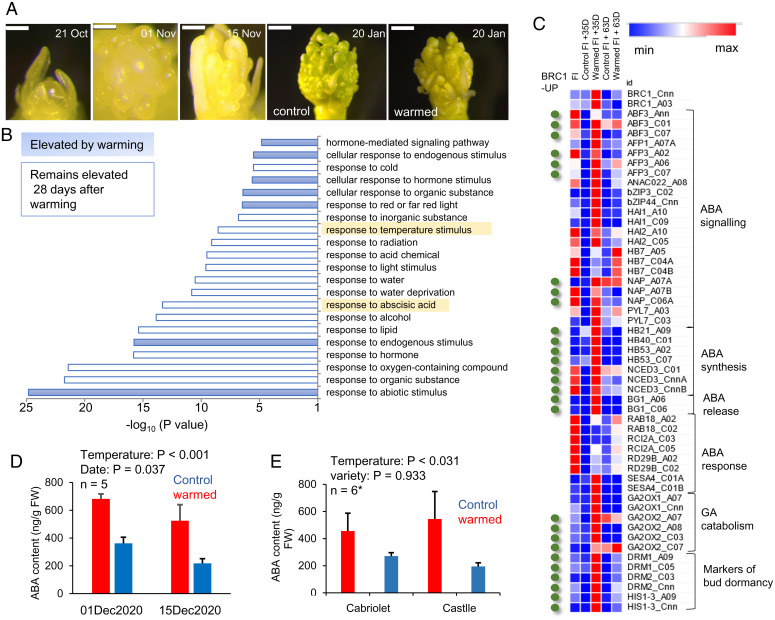
Winter warming induces dormancy and ABA accumulation in WOSR flowering buds. (*A*) Bud development on simulated growing season, with comparison to warmed plants in simulated January. Scale bar 200 µm except January samples (500 µm). (*B*) Gene expression changes between apices from simulated control growing season versus those after 4 wk of winter warming treatment. GO term analysis of warming-induced genes shows enrichment for temperature- and ABA-responsive gene expression. (*C*) Analysis of ABA-related gene expression induced by winter warming shows a substantial overlap with the previously identified *BRC1* bud dormancy regulon in *Arabidopsis* (16). Time points include the floral initiation (FI) prior to warming, after 4 wk of warming (control/warmed), and 4 wk after the cessation of warming (Control/recovery). (*D* and *E*) Winter warming in the field (*D*) and after 4 wk in a laboratory warm winter simulation (*E*) causes an increase in ABA levels in WOSR flower buds. Significance testing was via two-way ANOVA.

To understand the mechanism of warming induced growth delay during WOSR flower development we next used RNA sequencing to compare the transcriptomes of individual inflorescence buds from plants grown in the simulated growing season before and after 4 wk of winter warming, comparing expression to transcript levels at the floral transition prior to warming. At this stage warmed buds appeared developmentally delayed compared to control buds, but warmed buds remained floral (Fig. 2*A*). Changing the temperature during winter resulted in a substantial change to the flower bud transcriptome (Fig. 2 *B* and *C* and *SI Appendix*, Fig. S5). Interestingly, cold winter–induced genes were highly enriched for transcripts relating to the cell cycle, cell division, DNA replication, and chromatin (*SI Appendix*, Fig. S5). This chilling induction of cell division–related gene expression is consistent with the faster progression to flowering in control versus warmed plants occurring via cell proliferation. This class of transcripts was highly enriched for genes with the binding sites of the cell cycle regulator *MYB3R4* (15), and *MYB3R4* transcripts were also significantly elevated in cold winter buds compared to warm winter buds (*SI Appendix*, Fig. S5).

Gene Ontology (GO) term analysis of warm-induced genes indicated significant enrichment in the category “response to abscisic acid” and genes related to temperature signaling (Fig. 2*B*). These genes were enriched for *Arabidopsis* abscisic acid (ABA) response elements in their promoters (*SI Appendix*, Fig. S5) and included orthologs of the ABA biosynthesis gene *NCED3*. Previous work in *Arabidopsis* has shown that *NCED3* expression is controlled by BRANCHED1 (BRC1) via three homeodomain transcription factors, that is, HDZIP21, HDZIP53, and HD-ZIP40 (16), in dormant lateral buds. Interestingly, we found that in addition to *NCED3*, warming increased *BRC1* expression, the expression of all three *HDZIP*s, and a large number of known *BRC1*-regulated genes associated with the ABA response (Fig. 2*C*). This shows that in WOSR winter warming activates a well-known ABA-related bud dormancy module with conserved functions from *Arabidopsis* to woody perennials (17). To test the hypothesis that winter warming induces ABA accumulation, we measured ABA levels in individual WOSR floral buds in simulated warmed and control winters, and in warmed and control field plots. In both experiments ABA levels were significantly elevated by winter warming (Fig. 2 *D* and *E*), so we therefore concluded that winter warming delays flowering by inducing an ABA-related bud dormancy in WOSR via inhibition of cell proliferation.

**Fig. 3.**
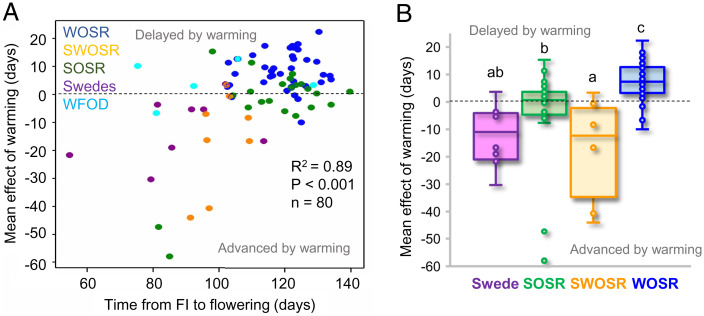
The effect of winter warming during flower bud development in a *B. napus* variety panel varies with crop type. (*A*) Warming causes a range of advances and delays to flowering time of individual varieties, with strong correlation between temperature response and time from the floral initiation (FI) to first flower opening under control conditions, as shown by R^2^. (*B*) *B. napus* responses to winter warming varied by crop type, with advances in flowering time in Chinese semiwinter varieties (SWOSR) and swedes, delays in WOSR, and no response in spring OSR (SOSR). Significant differences were determined by one-way ANOVA with a Fisher post hoc test at *P* = 0.05. Data for individual varieties were calculated from the mean of up to three replicate plants per variety.

To understand variation in winter bud dormancy in *B. napus* we grew a variety panel of mixed crop types (18), staggering sowing dates so that all lines passed through the floral transition in late autumn. Warming treatments were applied to flower buds for 4 wk using a heated glasshouse and compared to plants maintained in an unheated polytunnel (see *Materials and Methods*). A wide range in effects of inflorescence bud warming was observed in *B. napus*, from strong floral promotive effects in some varieties to delays of flowering in others (Fig. 3). The effect of warming was correlated with flowering time: late flowering lines were more likely to be delayed by warming and early flowering varieties were more likely to be advanced (Fig. 3*A*). Furthermore, responses were clearly separated by crop type. In WOSR winter warming of inflorescence buds almost universally delayed flowering, and in spring varieties varying the temperature had no effect on flowering time. In contrast, for Chinese semiwinter OSR and swedes, warming strongly promoted early flowering (Fig. 3*B*). Thus, we concluded that individual *B. napus* crop types have been bred to exhibit specific responses to temperature variation during flower bud development, and that the presence of bud dormancy in warm winters is genetically determined.

**Fig. 4.**
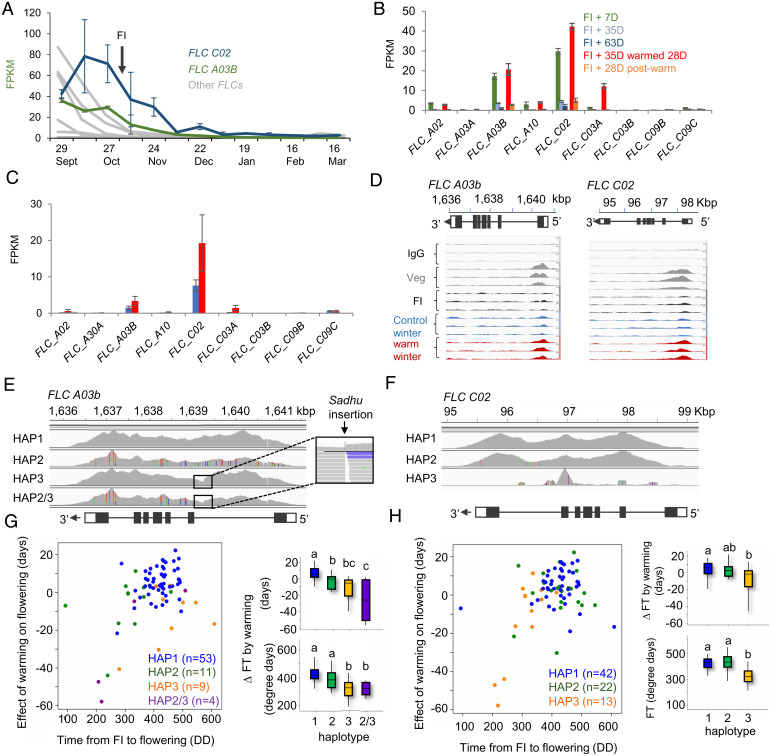
Two FLC-specific isoforms are associated with control of flower bud dormancy by chilling in WOSR. (*A*) Transcriptome analysis reveals that winter silencing of *FLC A03B* and *FLC C02* takes place after the floral initiation (FI) in WOSR in the field, using data from O’Neill et al. (2). Data shows the mean ± SE of three biological replicates. (*B*) Expression responses of WOSR FLCs to flower bud warming 7 d after floral initiation (FI) and during and after the indicated control and warming periods. Data show the mean ± SE of three biological replicates. (*C*) Transcript analysis of individual reproductive apices of WOSR apices in the field at the end of a 6-wk warming treatment (November 19, 2018 to January 3, 2019), showing continued expression of *FLC C02* and *FLC A03B*. Data show mean ± SE of three apices. (*D*) Chromatin immunoprecipitation for the active chromatin mark H3K4me3 at the expressed FLC genes from pools of shoot apices or flower buds from simulated control or warm winters, relative to input (IgG). Samples are 6 wk after sowing (Veg), at floral initiation (FI), or in flower buds after 4 wk of simulated warmed and control winters. Two biological replicates are shown. (*E* and *F*) Haplotype analysis at *FLC A03B* and *FLC C02* using exome capture (23) showing polymorphisms relative to the Darmor bzh version 4 reference sequence (24). (*G* and *H*) Association between *FLC* haplotype variation at *FLC A03B* and *FLC C02* and the effect of temperature on time from the floral transition to flowering. Relationship between the effect of temperature and time to flowering in degree days (DD) is shown plus the effect of each haplotype, taking into account variation between crop type by two-way ANOVA. ΔFT, change in flowering time caused by warming. Significant differences at *P* < 0.05 are shown, calculated by a Fisher exact test.

In *Arabidopsis* seasonal signaling pathways affect lateral bud development via association of FLOWERING LOCUS T (FT) and TERMINAL FLOWER 1 (TFL1) with *BRC1* (17, 19): variation at these genes also correlates with yield in *B. napus* (20, 21). Furthermore, *BRC1* is also a direct target of *FLC* (22). We previously showed that transcript abundance of two orthologs of *B. napus FLC*, *FLC A03B* and *FLC C02*, remain unaffected by vernalizing temperatures prior to the floral transition (2). Instead, expression of *FLC A03B* and *FLC C02* declines during winter chilling of developing flower buds, and this decline is prevented by winter warming in the simulated growing season and the field (Fig. 4 *A*–*C*). Chromatin immunoprecipitation for the active epigenetic mark H3K4me3 shows that this mark had already been lost from both *FLC*s at the floral transition but reappeared after winter warming, showing that warm weather in winter reverses the loss of active epigenetic marks at these loci (Fig. 4*D*). We compiled a list of flowering-related genes induced by winter warming that might affect bud behavior (*SI Appendix*, Table S1), which in addition to *FLC*s included several orthologs of *TFL1* and *MADS AFFECTING FLOWERING* (*MAF*).

We next identified haplotype variation at these loci from exome capture data (23) and explored the relationship with three traits: time from floral transition to flowering, time from floral transition to flowering after warming, and delay/advance in flowering caused by warming, measured in either calendar days or degree days. Variation at several loci showed some relationship to the effect of winter warming of flower buds on flowering time, but only variation at *FLC* remained significant after accounting for effect of crop type (*SI Appendix*, Table S2 and Dataset S1). We found three major haplotypes of *FLC A03B* (Fig. 4*E*): one corresponds to the Darmor *bzh* reference sequence (24) (a WOSR variety); HAP2 is characterized by several single-nucleotide polymorphisms (SNPs) relative to the Darmor *bzh* sequence, and a third haplotype (HAP3) which contains a *SADHU* transposon insertion in the first intron. Our set included four varieties with a haplotype resulting from a recombination event between HAP2 and HAP3. Transposon insertions are frequently associated with weak alleles of *FLC* (25). Lines containing the transposon insertion exhibited a range of floral development times but were never delayed by warming (Fig. 4*G*). For *FLC C02*, in addition to the Darmor reference haplotype (HAP1) there is one haplotype characterized by a small number of SNPs (HAP2), and one in which only mis-mapping reads aligned to the reference sequence, suggesting that *FLC C02* is deleted in HAP3 (Fig. 4*F*). This deletion has previously been reported to be associated with flowering time in *B. napus* (26). The lines carrying the deletion in *FLC C02* (HAP3) behaved differently to those with HAP1 and HAP2, requiring fewer degree days between the floral transition and flowering (Fig. 4*H*). Only one line with HAP3 was delayed by warming, showing that the *FLC C02* deletion is associated with time from floral transition to flowering. Taken together these results show that the effect of temperature on flower bud behavior in *B. napus* is under genetic control, and variation specifically at *FLC* isoforms which are affected by chilling after the floral transition correlates with the effect of temperature on flowering time from the floral transition to the first open flowers.

## Discussion

Much previous work on the timing of flowering in annual species has focused on understanding the mechanisms underlying control of the floral transition, although it has long been recognized that even *A. thaliana* has a two-stage floral transition. Here we show that in important winter annual crops winter flower bud dormancy also plays a role in the control of flowering time, in addition to the regulation of the duration of the vegetative phase. We conclude that WOSR exhibits bud dormancy in late autumn and early winter because warming delays development and causes maintenance of high ABA levels. Furthermore, warming inhibits growth-related gene expression and induces a transcriptional program associated with bud dormancy in other systems (16, 17). This is unlikely to be due to drought stress induced by warming because plants have access to ample water in the laboratory and field warming experiments, and warmed plants do not show signs of stress even after 4 wk of warming treatment (*SI Appendix*, Fig. S2), such as leaf loss. This contrasts with the effect of warming treatment applied later in winter which does accelerate flowering (2), presumably because by this stage bud dormancy is broken by chilling. We propose that this bud dormancy response can explain why substantial delays to the floral transition do not necessarily relate to large changes in the timing of flowering (2). Interestingly, even in *Arabidopsis*, mutations that substantially affect flowering time in laboratory experiments do not necessarily show large differences in flowering time in the field (27). Thus, control of flowering in winter annuals may more closely resemble that of perennials than summer annual species, which also show bud dormancy control via *FLC*-like genes and its partner SHORT VEGETATIVE PHASE (28–30). In perennial crops failure to break bud dormancy can result in yield losses via problems in bud break, flower abscission, floral organ, and fruit abnormalities (31). Here we show similar processes can be important in winter annual arable crops. Given that rapeseed crop models can be unreliable predictors of yield (32), including parameters for temperature effects on early floral development could improve their accuracy.

## Materials and Methods

### CER Growing Season Simulation.

We twice completed a full simulation of the 2016/2017 WOSR growing season in Norwich, UK, using data recorded at the farm weather station (Longitude, Latitude: +52.631, +1.179) in a Conviron BDW80 growth room with an ARGUS controller (Conviron) with 24 daily set points for temperature and photoperiod. Seeds were sown on simulated August 24, 2016 and plants harvested on simulated August 10, 2017. Plants were watered as required. After the floral transition warmed plants were transferred to a second identical chamber for 45 d where the temperature was increased by 10 °C. Plants were then placed in a single chamber for flowering and seed set in simulated winter, spring, and summer. Winter annual *Arabidopsis* accessions Lov-1, Var2-6, and Ull2-5 were grown using the same program and dissected weekly to visualize meristem morphology.

### Field Trials.

Field trials were conducted at the John Innes Centre experimental farm in Norwich, UK. WOSR Cabriolet seeds were drilled in twelve 6- × 1.2-m plots on August 20, 2020 with paired warmed and control plots. Plots were covered with Enviromesh to prevent insect ingress from September 2 to September 21. Plots were warmed from November l6 to December 18 as described previously (2). Plant heights were measured on March 19, 2021. Flowering time was scored as the appearance of the first open flower on each plant in each plot, with the percentage of plants in flower in each plot noted for each treatment. Field trial treatments are described in *SI Appendix*, Table S3. The trial was harvested on August 4, 2021 with yields normalized to 7% moisture measured by a Sinar GrainPro 6070 (Sinar Technology). Temperatures were measured with Tinytag TGP-4017 environmental dataloggers (Gemini Data Loggers Ltd) in the six warmed plots and four of the control plots.

### Diversity Set Analysis.

Ninety lines from the *B. napus* Diversity Fixed Foundation Set (18) were sown in a fully ventilated polytunnel in staggered fashion, cohorted by flowering time in autumn 2020. One plant of each line was confirmed by dissection as floral: then, 2 wk after the floral transition, three plants of each variety were transferred to a heated but unlit glasshouse maintained at 20 °C/16 °C day/night temperatures for 4 wk and compared to plants that remained in the polytunnel. Plants were then potted into 5-L pots randomized for flowering using a complete block design. Plants were scored for date to first flower opening and bud emergence using the BBCH (Biologische Bundesanstalt, Bundessortenamt und Chemische Industrie) scale (33). Accumulated thermal time was calculated as degree days calculated as hourly Σ(T − T_b_)/24 where T is the temperature in degrees Celsius and T_b_ is the base temperature, which was set to 3 °C (34).

### Gene Expression Analysis.

RNA was harvested from three single shoot apices and frozen in liquid nitrogen. RNA was extracted according to the manufacturer’s instructions using an EZNA plant RNA kit (Omega Bio-tek). The RNA samples were processed at Novogene using an Illumina NovaSeq 6000 to construct strand-specific libraries; 250- to 300-bp paired-end sequences with 23 to 36 million reads per sample were acquired and deposited at the National Center for Biotechnology Information (NBCI) under reference number PRJNA800835. In the field, material was harvested on January 3, 2019 at the end of warming. The clean reads were mapped to *B. napus* genome v4.1 (24) by HISAT2 v2.2.1 with default parameters (35). Gene expression levels and differential expressed genes were called by Cuffdiff v2.2.1 (false discovery rate ≤ 0.05, log_2_ fold change > 1) (36). Gene expression modules were measured by the WGCNA (weighted gene coexpression network analysis) package in R (37). For each module, the enriched motif in promoter regions (+100 bp to −2,000 bp) was identified by HOMER with default parameters (38).

### Chromatin Immunoprecipitation.

For each replicate of chromatin immunoprecipitation sequencing (ChIP-seq), floral buds from 10 plants were cut into 0.1- to 0.2-mm slices with a scalpel on ice. The sliced shoot apices were crosslinked under vacuum with 1% formaldehyde in a desiccator prefilled with ice during 15 min, and then quenched by replacing 2.5 mL of the crosslinking buffer by 2.5 mL of glycine under vacuum for an additional 5 min. The chromatin was extracted and then sheared by using a Universal Plant ChIP-seq kit (Diagenode, C01010152). Anti-H3K4me3 (Merck Millipore, 07-473) and normal rabbit IgG polyclonal antibody (Merck Millipore, 12-370) were immunoprecipitated with chromatin. Then de-crosslinked and purified DNA was submitted to library construction and sequencing (Novogene). Twenty-five to 38 million reads were acquired for each library, deposited at the NBCI Short Read Archive under reference PRJNA800835. The cleans reads were mapped to B. napus genome v4.1 by Bowtie2 v2.4.4 with default parameters (39). Additionally, PCR duplicates were marked and removed by MarkDuplicates of Picard tools v2.26.10. Then, the peaks of anti-H3K4me3 were normalized and visualized by bamCoverage of deepTools v2.3 and Integrative Genomics Viewer (IGV) v2.12.0, respectively (40, 41).

### Microscopy.

Single floral buds were manually dissected and imaged using a Leica M80 dissection microscope fitted with a Leica DFC295 digital camera.

### ABA Measurement.

Individual inflorescence buds were ground and extracted overnight at 4 °C with 99:1 isopropanol/acetic acid. d6-ABA was added as an internal standard. Supernatant was collected after centrifugation before drying in an evaporator. The dried extracts were resuspended in methanol and filtered through 0.22-μm Corning Costar Spin-X plastic centrifuge tube filters (Sigma-Aldrich). The solution was injected and analyzed on an ultraperformance liquid chromatography–mass spectrometry system.

## Supplementary Material

Supplementary File

Supplementary File

## Data Availability

Illumina sequence data have been deposited in NCBI (PRJNA800835) (42). Previously published data were used for this work (PRJNA309368) (43). All other study data are included in the article and/or SI Appendix.
