# Supplementary material for: Winter warming post floral initiation delays flowering via bud dormancy activation and affects yield in a winter annual crop
Source: Proc Natl Acad Sci U S A. 2022 Sep 19;119(39):e2204355119. doi: 10.1073/pnas.2204355119 (PMC9522361; doi:10.1073/pnas.2204355119)
Supplement: Supplementary File [file pnas.2204355119.sapp.pdf]

Supplemental Appendix for Lu et al. 2022

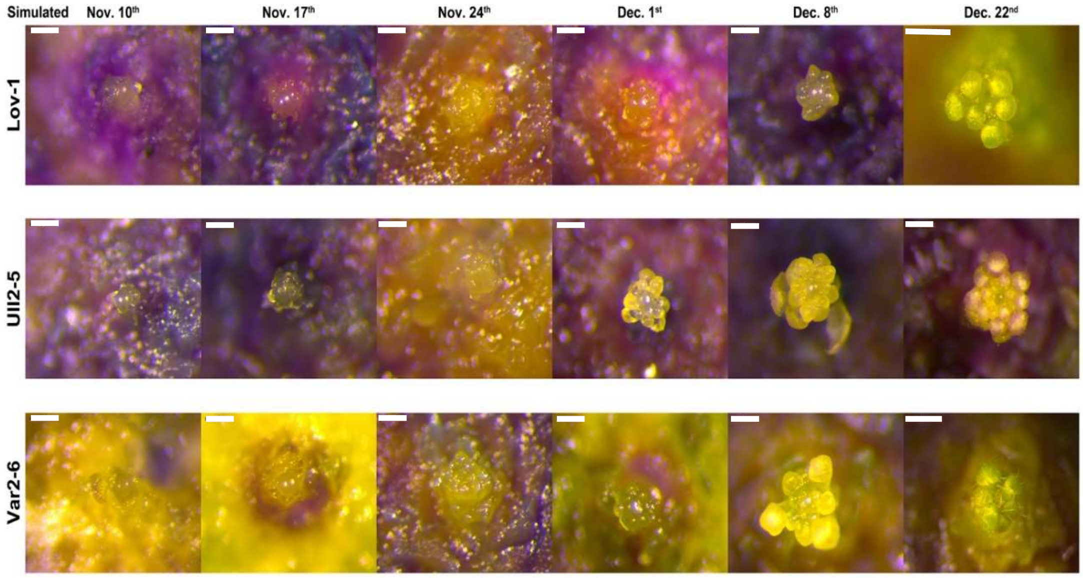

Figure S1. Autumn/winter development of the apical meristem of three Arabidopsis accessions with strong vernalisation requirements in a simulated Norwich, UK growing season, demonstrating the timing of floral development. Scale bars 200 µM, except for simulated December 22nd (500 µM).

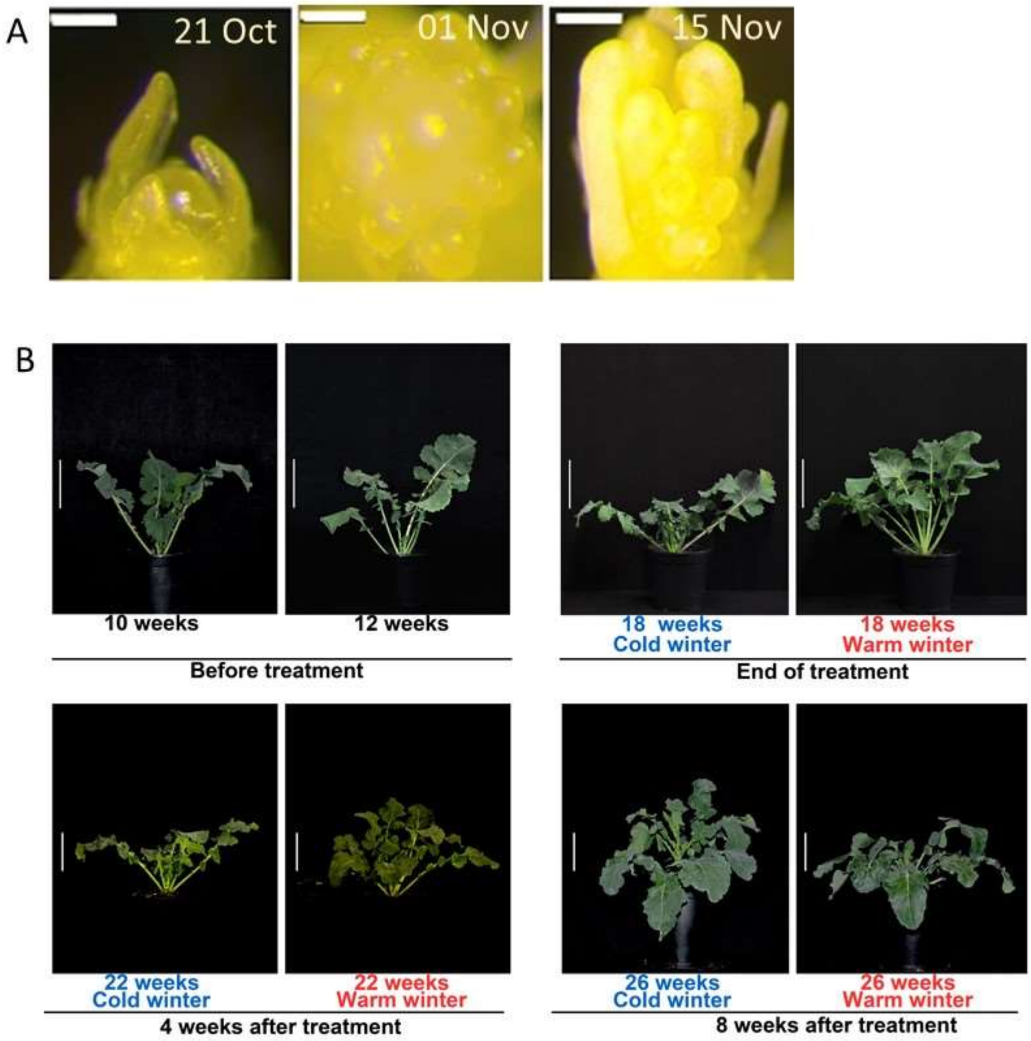

Figure S2. Developmental progression in the simulated winter annual growing season. A. The timing of the floral transition in the field is replicated in the whole growing season simulation. Data shows images of individual apices in the simulated Norwich UK 2016/17 growing season, with early floral development obvious in the first week of November. The floral transition timing is identical to that observed in the field, previously reported in (*2*). Scale bars 200 µM. **B**. Growth and development of winter OSR ‘cabriolet’ showing representative whole plants at the indicated timepoints after sowing and after control and warm winter treatments. Scale bars 10 cm.

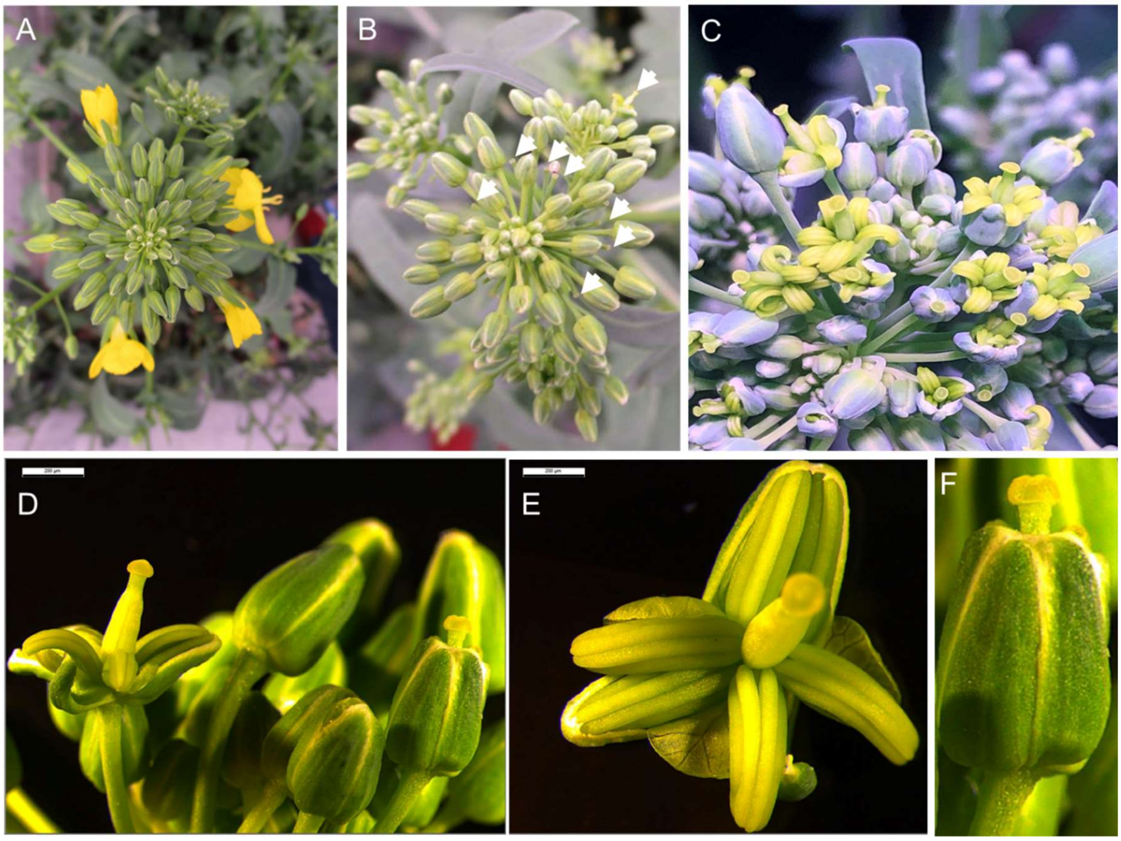

Figure S3. Example developmental consequences of warming WOSR floral buds in winter. A. Control WOSR variety Cabriolet inflorescence at the onset of flowering. B. Warmed inflorescence showing abnormal flowers and aborted flower buds indicated by the white arrows. C-E Flowers of WOSR variety Tapidor showing apetalous flowers opening before stamen maturity.

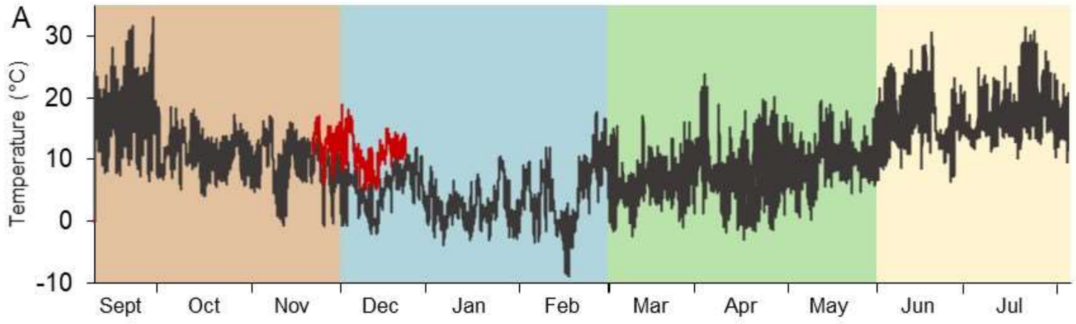

B

datalogger	H1	H2	H3	H4	H5	H6	C1	C2	C3	C4
Tmean (°C)	10.4	12.0	11.4	10.7	11.6	11.1	5.4	5.7	5.5	5.6
Tmax (°C)	18.6	20.4	18.2	18.9	20.3	18.1	15.8	14.6	13.8	15.3
Tmin (°C)	2.6	6.1	3.5	-0.1	4.6	3.3	-2.9	-2.1	-1.6	-2.6

Figure S4. Temperature profiles of control and warmed plots from the 2020/2021 field trial. A. Temperature data series from control (grey) and warmed (red) plots for the growing season at 30 minute resolution. Control data is from one datalogger except for the November/December temperature treatments where data is the mean of four dataloggers in four of six control plots. Warmed data is the average of 6 dataloggers in each of the warmed plots except for the last week during which one logger failed and was excluded from the analysis. **B**. Mean, maximum and minimum temperatures recorded by each datalogger. H -heated plot. C- control plot (4 of 6 control plots had dataloggers).

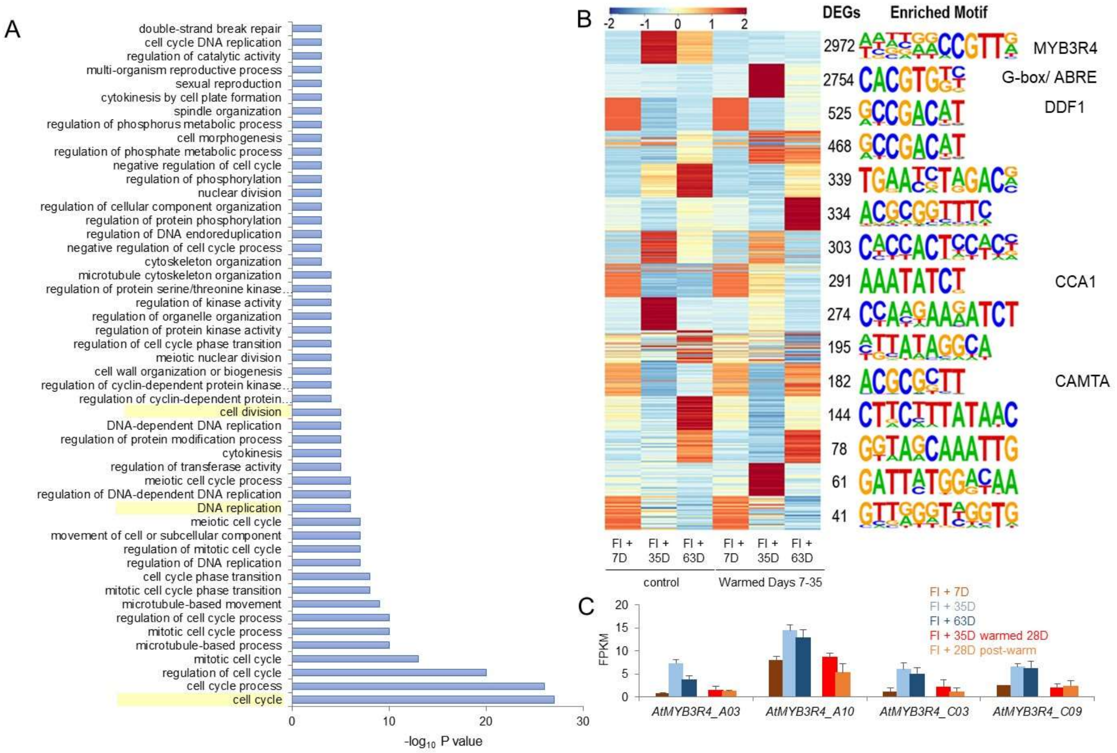

Figure S5. Chilling activates MYB3R4-regulated cell cycle gene expression in WOSR. A. GO- term analysis of chilling-induced genes in WOSR inflorescence buds, showing multiple categories related to cell cycle control and DNA replication. B. Cis-element analysis shows high enrichment for genes with *MYB3R4* binding sites in their promoters in chilling-induced genes. Red = high expression, blue low. FT- floral transition. C. Transcriptome analysis reveals that chilling increases expression of 4 *B. napus* orthologues of Arabidopsis *MYB3R4*.

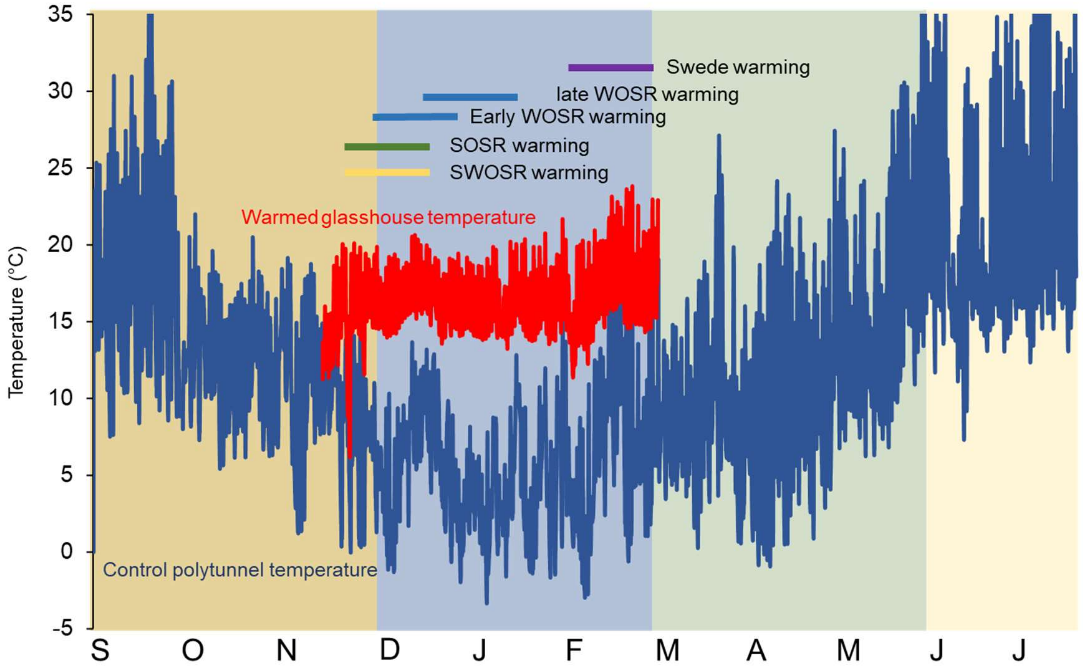

Figure S6. Temperature profiles for the *B. napus* variety panel warming experiment. All plants were grown in an unheated polytunnel in autumn 2020 (blue datalogger), with 3 of each genotype transferred to a heated but unlit glasshouse (red datalogger) 1 week after the floral transition for 4 weeks, while 3 control plants were retained in the polytunnel. The timing of warming treatments for the different crop types are shown, which are different due to the timing of the floral transition in each cohort. Note that floral transition times for WOSR are later than in the field due to warmer conditions in the polytunnel delaying vernalization.

Table S1. Identification of genes known for their role in plant phenology that are induced by warming in winter oilseed rape flower buds. Mean FPKM of significantly warm-induced genes are shown (q < 0.01) at the floral transition (FT) and in control or warmed flower buds.

***B. napus*_id**	**At_ID**	**Bn_synonym**	**FT mean**	**Control winter mean**	**Warm winter mean**	**log2 (fold _change)**
BnaC03g04170D	AT5G10140	*FLC_C03A*	1.4	0.3	12.1	5.29
BnaC02g43420D	AT5G65060	*MAF3_C02A*	11.2	0.6	10.3	4.03
BnaC02g00490D	AT5G10140	*FLC_C02*	29.8	4.5	42.3	3.22
BnaA06g23990D	AT5G65060	*MAF3_A06*	3.4	0.8	5.31	2.67
BnaA03g13630D	AT5G10140	*FLC_A03B*	17.2	3.6	20.6	2.51
BnaA05g01090D	AT2G46790	*PRR9_A05*	4.2	2.6	14.1	2.45
BnaC03g01440D	AT5G03840	*TFL1_C03*	55.7	5.8	28.3	2.37
BnaC09g04380D	AT5G24470	*PRR5_C09*	2.7	0.9	4.0	2.07
BnaA03g19970D	AT2G43010	*PIF4_A03*	0.3	0.7	2.9	2.04
BnaC07g29960D	AT5G24470	*PRR5_C07*	161.3	37.0	137.7	1.90
BnaAnng00810D	AT5G03840	*TFL1_Ann*	1.5	0.7	2.3	1.79
BnaC02g43470D	AT5G65060	*MAF3_C02B*	34.4	13.3	44.3	1.74
BnaC03g23970D	AT2G43010	*PIF4_C03B*	0.5	2.3	7.6	1.71
BnaA02g34510D	AT5G65060	*MAF3_A02*	20.0	7.3	22.9	1.64
BnaA03g39820D	AT5G60910	*FUL_A03*	46.3	31.7	95.8	1.60
BnaC02g02900D	AT5G03840	*TFL1_C02*	5.9	2.3	5.0	1.15

Table S2. Association between haplotype classification at *B. napus* loci linked to flowering time regulation and effects of winter warming, and time between the floral transition and first flower opening (BBCH60). P values for associations are given by 1-way ANOVA for time to flowering in calendar days, and for those that were significant (P < 0.01), by 2 way ANOVA with crop type to control for population structure. Significant P values are highlighted (* P < 0.01; ** P < 0.001).

**1. P-values for haplotype associations by 1-way ANOVA.**			
**gene**	**time to BBCH60 (days) warmed**	**time to BBCH60 (days) control**	**BBCH60 warmed – control (days)**
TFL1 A10	0.003*	0.002*	0.051
TFL1 Ann	0.002*	0.001**	0.026
TFL1 C02	0.001**	0.003*	0.003*
TFL1 Cnn	0.504	0.191	0.504
FT A02	0.012	0.045	0.017
FT C02	0.048	0.804	0.001**
FT A07	0.358	0.57	0.288
FT C06	0.001**	0.001**	0.004*
FLC A02	0.106	0.013	0.798
FLC A03a	0.818	0.826	0.478
FLC A30b	0.001**	0.001**	0.001**
FLC A10	0.211	0.522	0.102
FLC C02	0.001**	0.008*	0.001**
FLC C03a	0.007*	0.004*	0.108
FLC C03b	0.765	0.583	0.875
FLC C09a	0.001**	0.001**	0.063
FLC C09b	0.001**	0.001**	0.003*
MAF A06	0.871	0.536	0.307
MAF C08	0.276	0.311	0.294
MAF A02a/b*	0.126	0.028	0.492
MAF C02a/b*	0.168	0.518	0.068
FUL A03	0.223	0.119	0.603
**2. Significant P values for haplotype associations using 2-way ANOVA with crop type as co-variate**			
TFL1 C02	0.007*		
FT C06		0.005*	
FLC A30b	0.002*		0.001**
FLC C02	0.007*		0.006*
FLC C09b	0.006*	0.006*	

* These loci contain duplications of *MAF* genes as in Arabidopsis. Exome capture gave linked haplotype classifications for the two copies.

Table S3. Field Trial fertilizer and agrochemical input schedule.

Date	Fertiliser	Rate (kg/ha)	Total Used (Kg)	Total N applied (kg/ha)	Total Sulphur (SO_3) applied (kg/ha)
20/08/2020	Sulphan	275	20	26	38
01/03/2021	Sulfan	300	22.5	36	22.5
19/03/2021	Kieserite	100	7.5		50
19/03/2021	Extran	150	11.25	33.5	
12/04/2020	Extran	150	57	33.5	
**Application date**	**input**	**Product**	**Crop Growth Stage**	**Rate L/Ha**	**Total Used in L/Ha**
20/09/2020	Herbicide	Belkar		0.25	250ml
21/09/2020	Herbicide	Falcon		0.5	500ml
22/09/2020	Nutrition	Nutriphyte PGA		0.375	375ml
02/10/2020	Insecticide	Hallmark		75ml	38ml
12/10/2020	Insecticide	Hallmark		75ML	5.6ml
12/10/2020	Nutrition	Nutriphyte PGA		375ML	28.125ml
19/10/2020	Insecticide	Karis		75ml	21ml
19/10/2020	Insecticide	Biscaya		300ml	84ml
27/10/2020	Fungicide	Proline		0.32	160ml
27/10/2020	Nutrition	Photrel		3kg	1.5kg
25/11/2020	Herbicide	Astrokerb		1.7	225ml
25/11/2020	Insecticide	Hallmark		75ml	11.25ml
25/11/2020	Fungicide	Proline		0.32	128ml+56ml
25/11/2020	Nutrition	Photrel		3kg	1.2kg+520g
04/02/2021	Herbicide	Fox		0.75	112ml
04/02/2021	Adjuvant	Headland Fortune		1	150ml
19/03/2021	Nutrition	Yara Vita Photrel	Green Bud	2kg	1.12kg
19/03/2021	Fungicide	Tesoro		0.5	280ml
31/03/2021	Insecticide	Explicit	Yellow Bud	0.85kg	170g
31/03/2021	Nutrition	Yara Vita Photrel		2kg	400g
14/04/2021	Insecticide	Mavrik	Early-mid flower	0.2	90ml
14/04/2021	Fungicide	Pictor		0.5	250ml
14/04/2021	Nutrition	Yara Vita Photrel		1.5	750ml
14/04/2021	Nutrition	Magnesium Sulphate		5kg	2.5kg
30/04/2021	Insecticide	Mavrik	Late Flower	0.2	75ml
30/04/2021	Fungicide	Aviator Xpro		1	375ml
30/04/2021	Nutrition	Mg Sulphate		5kg	1.87kg
19/07/2021	Desiccant	Gallup 360		4	0.8
19/07/2021	Pod Shatter Prevention	Pod-Stik		1	0.2
